# The Hippo Signaling Pathway in Drug Resistance in Cancer

**DOI:** 10.3390/cancers13020318

**Published:** 2021-01-16

**Authors:** Renya Zeng, Jixin Dong

**Affiliations:** Eppley Institute for Research in Cancer and Allied Diseases, Fred & Pamela Buffett Cancer Center, University of Nebraska Medical Center, Omaha, NE 68198, USA; renya.zeng@unmc.edu

**Keywords:** Hippo pathway, drug resistance

## Abstract

**Simple Summary:**

Although great breakthroughs have been made in cancer treatment following the development of targeted therapy and immune therapy, resistance against anti-cancer drugs remains one of the most challenging conundrums. Considerable effort has been made to discover the underlying mechanisms through which malignant tumor cells acquire or develop resistance to anti-cancer treatment. The Hippo signaling pathway appears to play an important role in this process. This review focuses on how components in the human Hippo signaling pathway contribute to drug resistance in a variety of cancer types. This article also summarizes current pharmacological interventions that are able to target the Hippo signaling pathway and serve as potential anti-cancer therapeutics.

**Abstract:**

Chemotherapy represents one of the most efficacious strategies to treat cancer patients, bringing advantageous changes at least temporarily even to those patients with incurable malignancies. However, most patients respond poorly after a certain number of cycles of treatment due to the development of drug resistance. Resistance to drugs administrated to cancer patients greatly limits the benefits that patients can achieve and continues to be a severe clinical difficulty. Among the mechanisms which have been uncovered to mediate anti-cancer drug resistance, the Hippo signaling pathway is gaining increasing attention due to the remarkable oncogenic activities of its components (for example, YAP and TAZ) and their druggable properties. This review will highlight current understanding of how the Hippo signaling pathway regulates anti-cancer drug resistance in tumor cells, and currently available pharmacological interventions targeting the Hippo pathway to eradicate malignant cells and potentially treat cancer patients.

## 1. Introduction

As a powerful weapon to kill aberrantly fast-growing tumor cells, chemotherapy represents one of the most efficacious treatment strategies for the majority of cancer patients, immensely prolonging progression-free survival and improving quality of life. However, most cancers remain largely incurable, due to their early asymptomatic nature and late diagnosis, local and distant metastasis, and the development of therapeutic resistance. Although combinations of multiple therapeutic agents have become the paradigm for cancer therapy as initial solutions to the resistance against single-agent treatment, drug resistance continues to be a huge obstacle [1]. Till now, although a variety of mechanisms through which tumor cells develop resistance against anti-cancer agents have been defined, the exact biological processes remain incompletely understood. Improved therapeutic outcome and survival can be achieved from deciphering the biological determinants of drug resistance in tumors, which will guide the design of novel strategies to defeat drug resistance. Numerous studies have unraveled how the dysfunction or dysregulation of the Hippo signaling pathway in human cancer cells contributes to anti-cancer drug resistance and how these mechanisms might be exploited to clinically benefit cancer patients. Herein, we briefly review the key components of the Hippo pathway in both *Drosophila* and humans, as well as how the activation or inactivation of these molecules (not only YAP/TAZ, but also other components in the Hippo pathway) leads to drug resistance. We also summarize the pharmacological agents that enable the intensive anti-cancer efficiency by targeting the Hippo pathway.

## 2. Hippo Signaling Network in *Drosophila* and Humans

The Hippo signaling pathway was first discovered and determined as a key regulator of organ size in *Drosophila melanogaster*, following the identification of four tumor suppressors, Warts (Wts [2,3]), Salvador (Sav [4,5]), Hippo (Hpo [6,7,8,9,10]) and Mob-as-tumor-suppressor (Mats [11]). These proteins comprise the core kinase cascade of the *Drosophila* Hippo signaling pathway, in which activated Hpo phosphorylates and activates Wts-Mats complex [8,12]. During this process, Sav, phosphorylated by Hpo as well, facilitates the activation of Wts and Mats by functioning as a scaffold protein [8,9]. The key downstream target of Wts is a transcriptional co-activator Yorkie (Yki) [13]. Although lacking DNA-binding domains, Yki interacts with its DNA-binding partner Scalloped (Sd) to regulate gene transcription [14]. Phosphorylation of Yki on multiple sites is the major regulatory mechanism for its activity, particularly the phosphorylation on Ser168 by Wts. Ser168 phosphorylation mediates the binding of Yki to 14-3-3 proteins in the cytoplasm, promoting the cytoplasmic sequestration of Yki and transcriptional inactivation of its target genes [15,16,17]. Wts also phosphorylates Yki on two additional sites, Ser111 and Ser250, which restrict Yki nuclear localization independent of 14-3-3 proteins [18]. In contrast, when the Hippo pathway is off, Yki is translocated and accumulates in the nucleus, where it interacts with the transcriptional factor Sd to activate the expression of various genes, for example, *cyclin E* (a cell cycle regulator) and *diap1* (inhibitor of apoptosis), thus accelerating cell proliferation and diminishing cell apoptosis [13]. Hpo, Wts, Sav and Mats act as tumor suppressors and negatively control Yki activity, hence, loss-of-function of these proteins leads to tissue overgrowth and tumor formation [4,5,6,7,8,9,10,11,13,14,19]. 

Significantly, the functionality and networking of the Hippo signaling pathway are highly conserved across species, from insects to mammals. In humans, the hippo pathway is implicated in various cancer-associated physiological and pathological processes, including cancer stem cell self-renewal, tumorigenesis and anti-cancer drug resistance [20,21,22,23,24,25,26]. 

**Figure 1:**
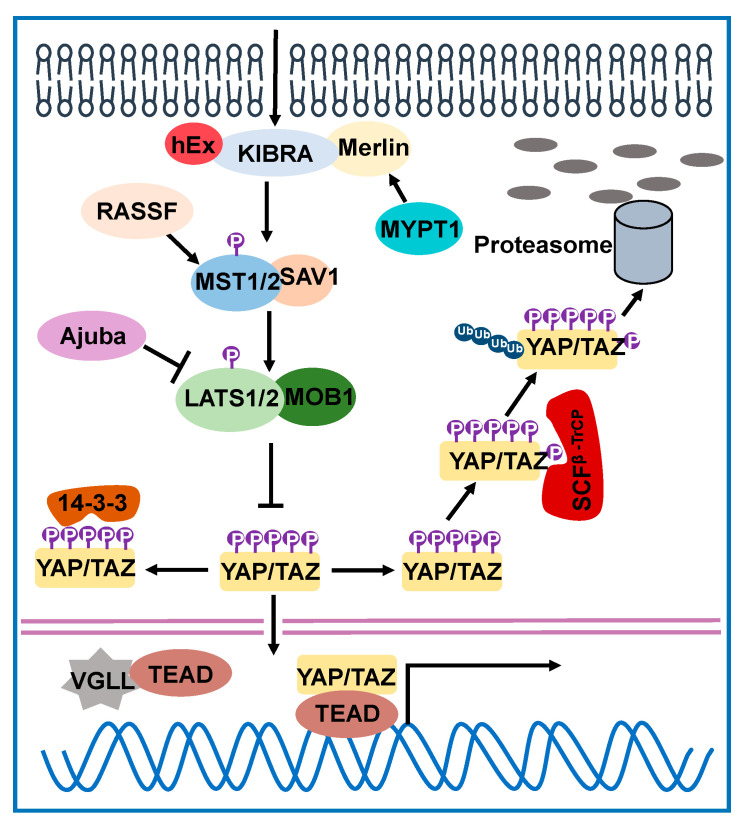
The Hippo signaling pathway in mammalian cells. Only regulators that have been implicated in drug resistance are shown. MST1/2, SAV1, LATS1/2, MOB1, YAP and TAZ compromise the core kinase cascade of the Hippo signaling pathway in mammalian cells. Activated MST1/2-SAV1 complex phosphorylates and activates LATS1/2-MOB1 complex, then the latter phosphorylates YAP/TAZ, leading to their cytoplasmic retention and ubiquitination-mediated degradation in proteasome. The activity of this kinase cascade is regulated by a variety of molecules at multiple levels. KIBRA, hEx and Merlin are upstream regulators that promote the activity of the kinase cascade. The activity of Merlin is regulated by MYPT1-PP1δ phosphatase, which dephosphorylates Merlin and activates it. RASSF family members (for example, RASSF1A and RASSF6) interact with MST1/2 and modulate its kinase activity. In contrast, Ajuba acts as a negative regulator through inhibiting the kinase activity of LATS1/2 and YAP/TAZ phosphorylation.

Core elements of the mammalian Hippo pathway include serine/threonine kinases, such as mammalian sterile 20-like kinase 1/2 (MST1/2; orthologs of *Drosophila* Hpo), and large tumor suppressor 1/2 (LATS1/2; orthologs of *Drosophila* Wts) (Figure 1). Salvador homolog 1 (SAV1) and MOB kinase activator 1A/B (MOB1A/B) function as the adaptor proteins of MST1/2 and LATS1/2, respectively. Like the *Drosophila* Hippo pathway, in mammalian cells the MST1/2-SAV1 complex phosphorylates and activates LATS1/2-MOB1A/B complex, and the activated LATS1/2-MOB1A/B complex phosphorylates and inactivates yes-associated protein (YAP) and transcriptional coactivator with PDZ-binding motif (TAZ), which are Yki orthologs in mammals [27]. Phosphorylation of YAP/TAZ on multiple sites by LATS1/2 promotes YAP/TAZ binding to 14-3-3 proteins and cytoplasmic retention. Amongst these sites, phosphorylation of Ser127 and Ser397 on YAP as well as Ser89 and Ser311 on TAZ are the dominant events in suppressing YAP/TAZ activities [28,29,30,31]. Phosphorylation of Ser397 on YAP (Ser311 on TAZ) primes phosphorylation of YAP on Ser400 (Ser314 on TAZ) by casein kinase 1 (CK1), leading to the recruitment of SCFβ -TrCP E3 ubiquitin ligase that mediates YAP/TAZ ubiquitination and proteasomal degradation [29,31]. When the Hippo signaling is off, YAP and TAZ are in activation status and shuttling to the nucleus to interact with TEAD transcriptional factors (TEAD1-4; orthologs of *Drosophila* Sd) and induce the expression of genes involved in cell proliferation, apoptosis, migration etc. Emerging studies have uncovered the diversity of the biological functions of the hippo pathway, ranging from organ size control, tissue homeostasis regulation, to disease development. Loss-of-function of the tumor suppressors (MST1/2, LATS1/2, SAV1 etc.) and activation of transcriptional coactivators YAP/TAZ (caused by protein overexpression and/or enhanced nuclear accumulation) play important roles in tumorigenesis and cancer-associated malignant features [27]. Due to genomic deletions or mutations (relatively rare), epigenetic silencing, or transcriptional and post-transcriptional regulations, tumor suppressors LATS1/2 and MST1/2 could undergo loss-of-function [32,33]. Hyperactivation of YAP/TAZ could be caused by other signaling pathways that contain oncogenic mutations (for example, the WNT, Hedgehog, and Notch pathways), other than direct mutations of the Hippo pathway components [34]. Nevertheless, amplification of YAP and TAZ, YAP gene fusions, and TAZ gene fusions caused by chromosomal translocation, although uncommon, have been documented in specific cancer types [35,36,37,38,39,40,41].

Strikingly, over the past decades, the mysteries of this kinase cascade-based network have been dramatically deciphered following the identification of numerous regulators of the Hippo pathway. These regulators impact the activity of the Hippo pathway at various levels, including kinases and adaptor proteins that directly regulate the activity of MST1/2 and LATS1/2, proteins associated with cell adhesions that modulate MST1/2, LATS1/2 and YAP activities, and a great many other proteins interacting with the canonical Hippo pathway components [42]. Furthermore, crosstalk between the Hippo pathway and other signaling pathways (e.g., TGF-β, Wnt, and G-protein-coupled receptor (GPCR) signaling) and critical cellular activities (e.g., metabolism and cell cycle) is well documented [43,44,45]. GPCRs, mechanical cues, actin remodeling regulators and signals transduced by the extracellular matrix and integrins can hypo-phosphorylate and activate YAP/TAZ through inactivating LATS1/2 or activating protein phosphatase 1 α (PP1A) [46,47]. These studies indicate the complexity of the Hippo pathway, but also provide a rich source of clues for Hippo pathway-targeted therapies. In this review, with the emphasis on the core components of the Hippo signaling pathway in human cells and their roles in anti-cancer drug resistance, we also discuss current pharmacological interventions targeting the Hippo pathway.

## 3. Hippo Signaling Pathway-Mediated Drug Resistance

### 3.1. Paclitaxel

The clinical introduction and application of paclitaxel marked a significant milestone in cancer treatment, particularly in ovarian and breast cancer. Through binding β-tubulin, this plant-derived agent (originally extracted from the Pacific yew tree, *Taxus brecifolia*) suppresses microtubule dynamic instability that is critical for the spindle formation during mitosis [48,49]. Disrupted mitotic spindles are not able to satisfy the spindle assembly check point, leading to mitotic arrest and eventually cell death, at least in in vitro models [50,51]. Resistance to paclitaxel can be attributed to a variety of reasons, including extensive drug efflux, inactivation of cell apoptosis machinery, and alterations of tubulin [52]. Huge efforts have been made to investigate the mechanisms underlying paclitaxel resistance, and recently the role of Hippo pathway in this process was defined (Table 1).

TAZ was firstly identified as a novel gene responsible for paclitaxel resistance in breast cancer via interacting with TEADs and transcriptionally activating connective tissue growth factor (CTGF) and cysteine-rich 61 (Cyr61) [80]. Overexpression of CTGF and Cyr61 renders resistance to paclitaxel-induced apoptosis through upregulating B-cell lymphoma-extra large (Bcl-xL, encoded by the *BCL2L1* gene), cellular inhibitor of apoptosis protein 1 (cIAP1) and X-linked inhibitor of apoptosis protein (XIAP) (they are anti-apoptotic proteins and function to inhibit the cell death induced by anti-cancer drugs), following the activation of the integrin αvβ3-ERK1/2 MAPK pathway or integrin-NF-κB pathway [97,98,99]. Interestingly, the activity of TAZ in response to paclitaxel treatment appears to be determined by its phosphorylation status. Upon paclitaxel treatment, independent of the hippo signaling pathway, the phosphorylation of TAZ is directly catalyzed by cyclin-dependent kinase 1 (CDK1) at six amino acids (Ser90, Ser105, Thr285, Thr175, Thr326 and Thr346), leading to TAZ degradation by proteasomes and abolishing TAZ-induced paclitaxel resistance [81]. Hence, it is possible the final consequence of the conflict between CDK1-mediated TAZ inactivation and TAZ-induced paclitaxel resistance is that when TAZ is overexpressed in tumor cells and limited CDK1 molecules are not sufficient to completely phosphorylate and inhibit TAZ, then activated TAZ will cause paclitaxel resistance [81]. However, convincing experimental evidence is required to support this hypothesis. 

Furthermore, it has been documented that TAZ is required for the metastatic capability and chemoresistance of prospective breast cancer stem cells (BCSCs), which are remarkably protected from apoptosis induced by paclitaxel and doxorubicin [82]. TAZ is highly expressed and active in BCSCs, compared with their differentiated breast cancer cell progeny that have lost chemoresistance and metastatic features. Of note, a more pronounced paclitaxel- and doxorubicin-induced cytotoxicity was observed in TAZ-depleted BCSCs, in contrast to BSCS controls, revealing that TAZ contributes to drug resistance in breast cancer [82]. Nonetheless, little is known about the downstream effectors of TAZ associated with drug resistance in breast cancer. A possible mechanism has emerged from another study on BCSCs, in which TAZ overexpression in BCSCs increased the activity of multidrug resistance (MDR) proteins [83]. MDR proteins are crucial cell membrane transporters that mediate drug efflux and reduce intracellular drug concentration, thus promoting anti-cancer drug resistance. However, more details regarding the mechanisms by which TAZ contributes to paclitaxel resistance will need to be pinpointed in future research. 

Likewise, YAP renders resistance to paclitaxel and cisplatin in multiple ovarian cancer cell lines. Overexpressing YAP-S127A (which lacks a major LATS1/2 phosphorylation site and accumulates in the nucleus) in cells with low baseline YAP activity confers resistance to paclitaxel and cisplatin, whereas YAP knockdown in cells with higher YAP activity increases sensitivity to paclitaxel and cisplatin [53]. 

Loss-of-function of LATS1/2, the YAP/TAZ upstream kinase, is also implicated in paclitaxel resistance. For example, siRNA-induced LATS1/2 knockdown in HeLa cells and paclitaxel-sensitive ovarian cancer cells significantly attenuates cell death stimulated by paclitaxel treatment, indicating the functional role of LATS1/2 in mediating paclitaxel-induced cell apoptosis [86,87]. Notably, alterations of LATS1/2 expression in tumor cells can be modulated by microRNAs (miRs). In ovarian cancer, the miR-363 level is elevated in paclitaxel-resistance cells, and introduction of miR-363 into paclitaxel-sensitive cells enhances paclitaxel resistance by suppressing LATS2 expression and promoting YAP nuclear accumulation [87]. The loss of LATS1 also induces a protective effect against paclitaxel in non-small cell lung cancer (NSCLC) cells [88].

Ras association domain family 1 isoform A (RASSF1A) is an upstream regulator in the Hippo pathway. It interacts with MST1/2 to modulate mitotic progression and cell apoptosis [100,101,102]. RASSF1A is epigenetically silenced by promoter DNA methylation in more than 50% of all solid tumors, and loss of it correlates with the acquisition of paclitaxel resistance in ovarian cancer and breast cancer patients [54,90,91]. Cells pretreated with DNA methyl transferase (DNMT) inhibitors, zebularine and RG108, exhibited an enhanced sensitivity to paclitaxel, revealing a potential therapeutic strategy by restoring RASSF1A levels [90].

Human Expanded (hEx) is the mammalian ortholog of Ex in *Drosophila* that abrogates the activity of Yki by binding to it and translocating it out of the nucleus [103]. In human breast cancer cells, hEx inhibits cell proliferation and sensitizes cells to paclitaxel, while knockdown of hEx confers paclitaxel resistance [92]. 

It is noteworthy that the intratumoral concentration of paclitaxel was found to be below the concentrations required to induce sustained mitotic arrest in vitro [104]. Tumor cells in the lower and clinically relevant concentrations of paclitaxel (low nM range) proceed through mitosis without substantial delay and divide their chromosomes on multipolar spindles, causing chromosome missegregation, the generation of aneuploid progeny and cell death [104,105]. Research has unveiled the aneuploidy caused by low concentrations of paclitaxel is associated with the dissociation of p55CDC (also known as cell division cycle 20 (CDC20))-mitotic arrest deficient 2 (Mad2) or p55CDC-BUB1-related protein kinase (BubR1) complexes, which are pivotal components of the spindle checkpoint [105]. And high levels of mitotic Aurora kinase A (AURKA) and its cofactor targeting protein for Xklp2 (TPX2) are also essentially implicated in this distinct mitotic phenotype in response to paclitaxel at low concentrations [106]. The findings achieved by our lab and other scientific research teams have identified the links of the Hippo components (for example, YAP, the WW domain-containing protein KIBRA, and Merlin) with BubR1 or AURKA in regulating cell cycle [107,108,109,110]. Interestingly, as is demonstrated in the studies described above, knockdown of YAP/TAZ or overexpression of LATS2, RASSF1A or hEx is able to sensitize cancer cells to paclitaxel at low concentrations [53,54,80,82,83,87,90,92]. However, how the deregulation of the Hippo components in cancer cells accelerates paclitaxel-induced cell death, and whether the occurrence of aneuploidy in response to low and clinically relevant concentrations of paclitaxel is enhanced upon the deregulation of Hippo pathway remain to be defined. Future studies investigating these mechanisms might build a consolidated pre-clinical basis for targeting the Hippo pathway to overcome paclitaxel resistance.

### 3.2. Cisplatin

Regarding the chemotherapy regimens for ovarian cancer patients after cytoreductive surgery, a platinum compound (for example, cisplatin or carboplatin) is always administrated together with paclitaxel as the first-line treatment. Once these platinum compounds enter the cells, they form bonds with DNA and cause DNA damage, blocking cell division and resulting in apoptosis [111]. Similar to resistance to paclitaxel, resistance to cisplatin also limits the benefit that patients can obtain from the treatment. The tumor suppressor Merlin (encoded by neurofibromatosis type 2 gene (NF2)) is required for the activation of the Hippo pathway by recruiting LATS1/2 to the plasma membrane and enhancing its phosphorylation by the MST1/2-SAV1 complex [112,113,114]. Dephosphorylation of Ser518 on Merlin is particularly important for its function [115]. Myosin phosphatase MYPT1-PP1δ, formed by the catalytic subunit PP1δ and the regulatory protein MYPT1, can dephosphorylate Merlin at Ser518 and activate it [112]. Interestingly, MYPT1 is often downregulated in ovarian cancers, which is associated with worse overall survival and lower platinum sensitivity [93] (Table 1). Either MYPT1 deletion or downregulation by miR-130 leads to significantly increased activity of YAP/TAZ (probably through attenuating Merlin dephosphorylation and activation), evaluated as the decreased ratio of phosphorylated YAP-Ser127 over total YAP, as well as elevated nuclear accumulation of YAP/TAZ. Via accelerating YAP/TAZ activity, MYPT1 downregulation potentiates platinum compound resistance and cancer stem cell properties. Of importance, YAP inhibition by either peptide 17 or verteporfin dramatically restores platinum compound sensitivity and reduces stemness [93]. 

Ajuba LIM proteins (Ajuba, LIMD1 and WTIP) act as negative regulators of the Hippo pathway. They interact with LATS1/2 and SAV1 to antagonize the phosphorylation of YAP and TAZ [116]. Overexpression of Ajuba is observed in cervical cancer and high levels of Ajuba promote cisplatin resistance in cervical cancer by upregulating its downstream mediators YAP and TAZ [94].

Cisplatin is also an efficacious therapeutic agent for nasopharyngeal carcinoma and prostate cancer patients. Overexpression of TAZ was reported to be positively associated with cisplatin resistance and epithelial-mesenchymal transition (EMT) in nasopharyngeal carcinoma [84]. Similarly, TAZ depletion is able to reverse EMT phenotypes and sensitize cells to cisplatin treatment. Moreover, in prostate cancer, MST1 was found to interact with heat shock protein 70 (Hsp70), and the functional consequences of this interaction are the degradation of MST1 and cisplatin resistance [85]. In contrast, overexpression of MST1 augments cisplatin-induced apoptosis in prostate cancer cells. 

On the contrary, in head and neck squamous cell carcinomas (HNSCC) cell lines, YAP knockdown by siRNA surprisingly enhances proliferation, migration and cisplatin resistance [55]. An explanation is that the oncogenic or tumor suppressing role of YAP might be molecular and tissue context-dependent, and the status of p53 family members (such as p53, p63 and p73) seems to determine YAP function as well [55]. 

### 3.3. Doxorubicin

Doxorubicin is an anthracycline drug commonly used in the treatment of breast cancer, lung cancer, and ovarian cancer by disrupting topoisomerase II-mediated DNA repair, and promoting the damages of free radicals to multiple cellular structures [117]. Overexpression of YAP/TAZ potentiates doxorubicin resistance in hepatocellular carcinoma (HCC) and conversely YAP/TAZ downregulation by RNA interference restores doxorubicin sensitivity [56,57] (Table 1). Mechanistically, YAP induces resistance against doxorubicin partially by activating the mitogen-activated protein kinase (MAPK) pathway, while TAZ activates interleukin-8 (IL-8) transcription to induce resistance. A small molecule, C19, with remarkable inhibitory effects on the Hippo-YAP, Wnt and TGF-β pathways, was demonstrated to attenuate doxorubicin resistance in vitro and in vivo [58]. In contrast, another study reported that both YAP and p53 are activated in doxorubicin-treated HCC cells and overexpressing wildtype YAP in these cells induces doxorubicin sensitivity [59]. This research also showed YAP improves apoptosis by binding to the p53 promoter and enhancing p53 expression, and p53 positively promotes YAP expression by binding to the YAP promoter, suggesting a role for p53 in regulating YAP function [59]. As p53 is frequently mutated and inactive in tumor cells, determining whether YAP overexpression promotes doxorubicin sensitivity in p53 mutant tumor cells will be necessary to elucidate its function in doxorubicin responsiveness.

The tumor suppressor RASSF6 (another member in the RASSF family, along with RASSF1A), which is frequently downregulated in bladder cancer, regulates doxorubicin sensitivity via the Hippo pathway [95]. RASSF6 overexpression augments doxorubicin-triggered cell apoptosis by negatively regulating YAP and the pro-apoptosis protein Bcl-xL.

### 3.4. 5-Fluorouracil (5-FU)

5-FU is a fluoropyrimidine compound routinely used in treating colorectal cancer, breast cancer and pancreatic cancer either as a single agent or in combination with other chemotherapeutic drugs. The misincorporation of 5-FU into RNA and DNA structures and its inhibition of the nucleotide synthetic enzyme thymidylate synthase are the major mechanisms of its cytotoxicity [118]. An elevated YAP level is noted as a major change in 5-FU resistant colorectal cancer cells, in comparison with nonresistant cells, and YAP knockdown enhances 5-FU toxicity in vitro and in vivo [60] (Table 1). Indeed, tumor YAP expression is positively correlated with the survival rate of colon cancer patients, and epidermal growth factor receptor (EGFR)/YAP signaling plays a critical role in promoting 5-FU resistance [62]. In line with these observations, the synergistic combination of verteporfin and AG1478, an inhibitor of EGFR, was observed to reverse 5-FU resistance in cultured cells and mice. Other colon cancer research has shown the c-Yes/YAP axis drives 5-FU resistance through the combined acquisition of cell quiescence and stemness [61]. Collectively, these studies support the strategy of targeting YAP to overcome 5-FU resistance in colorectal cancer.

Other possibilities to enhance 5-FU cytotoxicity are overexpression of Merlin and LATS2. Merlin re-expression in glioma cells confers 5-FU sensitivity, likely through downregulating cIAP1 and cIAP2 that inhibit caspase activity and apoptosis [96]. Overexpression of LATS2 in combination with 5-FU treatment elevates the expression mitochondrial elongation factor 1 (MIEF1) via c-Jun N-terminal kinase (JNK) pathway, thus amplifying fatal mitochondrial stress [89]. 

### 3.5. Gemcitabine

Gemcitabine constitutes the backbone of standard pancreatic cancer treatment regimens; however, gemcitabine resistance remains a conundrum and partly accounts for the very low five-year survival rate of patients with this cancer type. One study revealed that overexpression of YAP contributes to EMT and gemcitabine resistance in pancreatic cancer cells, partly due to the activation of AKT [63] (Table 1). YAP’s role in gemcitabine resistance is also regulated by miRNAs. The hyperactivation of YAP can be induced by miR-181c-mediated downregulation of its upstream components MST1, LATS2, MOB1 and SAV1, and the downstream effectors of YAP (for example, Bcl-xL) abrogate gemcitabine-induced cell death [64]. MiR-873 downregulation in triple-negative breast cancer (TNBC) promotes gemcitabine resistance through elevating the level of zinc finger E-box binding homeobox 1 (ZEB1), which interacts with YAP to activate gene transcription [65,66]. Taken together, these studies suggest YAP is a key player in gemcitabine resistance, and consequently, an attractive target to augment gemcitabine cytotoxicity. Indeed, YAP inhibition by verteporfin and gemcitabine synergistically eradicate cells that were formerly resistant to gemcitabine [67].

Paradoxically, a study found that Hippo inactivation sensitizes pancreatic cancer cells to gemcitabine both in vivo and in vitro [119]. Diverse pancreatic cancer cell lines become temporarily resistant to gemcitabine at high-density conditions that trigger the activation of the Hippo signaling pathway and YAP inactivation. In contrast, replating cells at low confluence reestablishes the gemcitabine sensitivity, accompanied with an elevated nuclear localization of YAP. Interestingly, activated YAP diminishes gemcitabine efflux by downregulating mRNA levels of cell membrane transporters, such as ATP-binding cassette (ABC) transporters. Moreover, YAP also downregulates the mRNA for cytidine deaminase, which catalyzes the enzymatic conversion of gemcitabine to its inactive uracil metabolite (dFdU) [119]. These findings complicate comprehension of the role of Hippo signaling in driving gemcitabine resistance, and future studies will be required to shed more light on it. These future studies might use immunocompetent mice to provide more answers to the exact role of YAP in gemcitabine resistance while taking the crosstalk between the Hippo pathway and tumor microenvironment or immune system into account. 

### 3.6. EGFR Inhibitor or Anti-EGFR Antibody

Pharmacologically inhibiting EGFR by specific tyrosine kinase inhibitors (TKIs) has been proven highly successful in treating lung cancer patients harboring EGFR-activating mutations. However, lung cancer still ranks the first among causes of cancer-related death in the United States [120], and the emergence of primary or acquired resistance severely hampers the response to EGFR-TKIs in almost every patient [121]. Mounting studies have indicated that YAP overexpression enhances resistance to EGFR-TKIs (like the first-generation inhibitors gefitinib and erlotinib, second-generation inhibitor afatinib, and third-generation inhibitor osimertinib) in multiple lung cancer cell lines; whereas YAP knockdown restores EGFR-TKI sensitivity [68,69,70,71] (Table 1). Addition of YAP inhibitor verteporfin phenocopies the effect of YAP knockdown [68,69]. Consistently, YAP downregulation-induced chemosensitivity to EGFR-TKIs was also detected in ovarian cancer and colorectal cancer cells expressing EGFR [72,73].

Investigations of YAP/TAZ downstream targets in mediating EGFR-TKIs resistance have defined possible molecular mechanisms. Growth factor amphiregulin (AREG) is an EGFR ligand and a target of YAP and TAZ. YAP/TAZ transcriptionally induce the overexpression and secretion of AREG, which mediates the malignant behaviors of mammary epithelial cells, such as proliferation and migration [122]. Secreted AREG is able to activate EGFR signaling, functioning as a bridge connecting the Hippo and EGFR signaling pathways. It is of interest to note that, by binding and activating EGFR, both AREG and transforming growth factor α (TGFα) induce the dephosphorylation of LATS1, MOB1 and YAP in the Hippo signaling pathway, thus activating YAP that drives the proliferation and migration of cervical cancer cells [123]. Activated YAP triggers the amplification of EGFR, AREG and TGFα, and therefore a positive loop is formed that promotes cervical cancer progression. 

### 3.7. HER2 Inhibitor

Consistent with the functions of YAP/TAZ in contributing to tumor resistance against EGFR-TKI therapies, these proteins are also required for the elastic modulus-dependent response to lapatinib, an FDA-approved human epidermal growth factor receptor 2 (HER2)-targeted kinase inhibitor. In this research, HER2-amplified breast cancer cells show greater resistance to lapatinib on rigid matrices in vitro, suggesting microenvironment stiffness modulates lapatinib resistance [74] (Table 1). Either YAP/TAZ knockdown or disruption of the TEAD-YAP interaction with verteporfin enables the improvement of lapatinib sensitivity [74]. 

### 3.8. CDK4/6 Inhibitor

Uncontrolled cell proliferation is a hallmark of malignancies [124]. The cell cycle is characterized by extremely organized regulation, in which cyclin-dependent kinases (CDKs) and their cyclin partners exert critical functions. Thereinto, CDK4 and CDK6 act in complex with D-type cyclins (cyclin D1, D2 and D3) to drive cell cycle progression from G0 or G1 phase to S phase in response to mitogenic stimulation [125,126]. Dysregulated cyclin D-CDK4/6 complexes lead to intensive cell growth, therefore accelerating tumor development in some cancer types that are particularly dependent on cyclin D-CDK4/6 activities [127,128]. As a consequence, these tumor cells are considered susceptible to CDK4/6-targeted inhibition. Strikingly, three selective CDK4/6 inhibitors (palbociclib, ribociclib, and abemaciclib) have acquired FDA approval for the treatment of patients with hormone receptor (HR)-positive and HER2-negative advanced or metastatic breast cancer [129]. However, the success of these treatments is tremendously restricted because of drug resistance, and YAP/TAZ activation provides an explanation. A genomic analysis of estrogen receptor-positive (ER+) breast cancer identified that loss of FAT1 causes Hippo signaling pathway suppression and subsequent YAP/TAZ activation [75]. Activated YAP/TAZ is accumulated on the CDK6 promoter and facilitates CDK6 transcription. Therefore, elevated CDK6 expression confers resistance to CDK4/6 inhibitors in breast cancer cells with FAT1 inactivation. Noteworthy, it is also hypothesized that other oncogenic alterations in the Hippo signaling pathway, for example NF2 loss, potentiates the resistance to CDK4/6 inhibitors, and it is necessary that these possibilities be further examined. 

### 3.9. RAF and MEK Inhibitors

Many cancers are characterized of the oncogenic activation of the RAS-RAF-MEK-ERK signaling pathway (also known as MAPK pathway). This pathway serves to transmit extracellular proliferation signals to the nucleus, thus promoting the transcription of genes that drive tumorigenic activities (e.g., cell growth and metastasis) and oncogenic transformation [130]. Constitutive activation of the MAPK pathway is mainly caused by the somatic activating mutations in RAS and BRAF, making them druggable therapeutic targets with great promise. Several inhibitors targeting BRAF and MEK have been deployed in patients with metastatic NSCLC harboring BRAF V600E mutations [131], patients with unresectable or metastatic melanoma harboring BRAF V600E/K mutations [132] and patients diagnosed with anaplastic thyroid cancer carrying BRAF V600E mutations [133]. However, therapeutic resistance arises and leads to transient and incomplete responses. 

Through a pooled short hairpin RNA (shRNA) screen in human NSCLC cells carrying BRAF V600E mutations, YAP was defined to act as a parallel survival input to promote resistance to BRAF and MEK inhibition by transcriptionally upregulating the expression of anti-apoptotic protein, Bcl-xL [76] (Table 1). YAP silencing is able to enhance sensitivity to vemurafenib (a BRAF inhibitor) and trametinib (a MEK inhibitor) in a variety of tumor cells carrying the BRAF V600E mutant, without affecting vehicle-treated tumors. Accordingly, YAP knockdown also enhances response to trametinib in KRAS-mutant NSCLC and pancreatic cancer cells [76]. Together, these findings suggest that YAP regulates the response to BRAF- and MEK-targeted therapy. This is further confirmed by the analysis of YAP levels in human tumor specimens collected from patients with NSCLC and melanoma encoding BRAF V600E mutations, NSCLC patients with KRAS mutations, and NSCLC patients with wild-type BRAF and KRAS [76]. Higher levels of YAP were detected in the majority of NSCLC and melanoma tumors harboring BRAF V600E and NSCLC tumors with KRAS mutations, compared to tumors expressing wild-type BRAF and KRAS [76]. Elevated YAP levels were also observed in tumors from patients with acquired BRAF or MEK inhibitor resistance, in comparison to the matched pretreated specimens, revealing that elevated YAP levels may limit clinical efficacy of BRAF and MEK inhibitors [76]. 

Strikingly, vemurafenib-resistant melanoma cells harboring BRAF V600E mutation display substantial increase in actin stress fiber formation, reduced apical actin pool and remarkably flattened cell shape in the presence of prolonged vemurafenib [77]. This actin cytoskeletal remodeling upon vemurafenib treatment increases YAP/TAZ nuclear localization and activation, which promote drug resistance and cancer cell survival. In line with the previous study, this research provides a verification that YAP/TAZ knockdown reduces vemurafenib-resistant cell viability [77]. It also reveals that constitutively active YAP contributes to vemurafenib resistance probably through its downstream effectors, such as E2F transcription factor 1 (E2F1), EGFR and c-MYC [77]. 

The MEK inhibitor trametinib is able to inhibit tumor cell growth in neuroblastoma with active ERK functions, suggesting a therapeutic treatment option for neuroblastoma patients [78]. However, extending trametinib use in neuroblastoma is challenged by the fact that constitutively active YAP overexpression induces resistance against trametinib in MAPK pathway-activated neuroblastoma cells via transcriptional activation of E2F and MYCN [79]. Conversely, loss of YAP sensitizes neuroblastoma cells to trametinib, implying that combinatorial inhibition of MEK and YAP signaling could be a therapeutic strategy for neuroblastoma patients with hyperactivated MAPK signaling. 

## 4. Targeting the Hippo Pathway

### 4.1. Verteporfin

**Figure 2:**
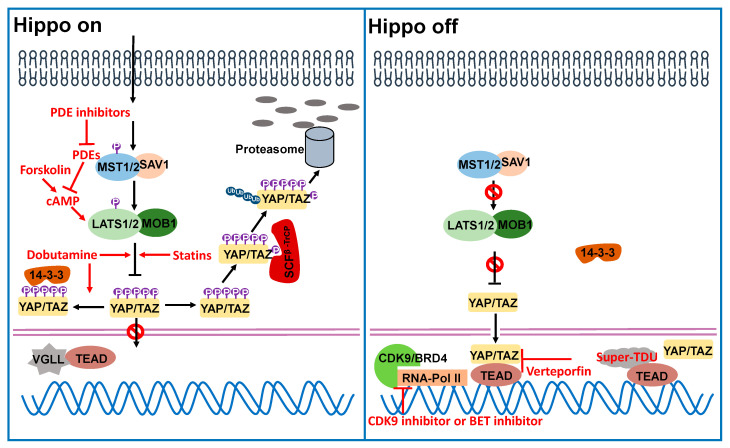
Targeting the Hippo signaling pathway. Both forskolin and PDE inhibitors elevate the cellular level of cAMP, which facilitates LATS1/2 kinase activity, and consequently leads to YAP phosphorylation, cytoplasmic accumulation and degradation. In addition, dobutamine and statins also induce the phosphorylation of YAP/TAZ. Verteporfin and Super-TDU act as YAP/TAZ inhibitors through blocking the physical interaction between YAP/TAZ and TEADs. The transcription of YAP/TAZ target genes can be impeded by CDK9 inhibitors and BET inhibitors, which block the CDK9 or BRD4-involved transcription process.

The photosensitizer verteporfin, activated by non-thermal laser light to selectively damage neovascular structures, is clinically approved in photodynamic therapy for neovascular macular degeneration [134,135]. In a drug library-based screening, verteporfin was identified as one of the top hits that were capable of inhibiting the physical interaction between YAP and TEADs [136] (Figure 2 and Table 2). Additionally, in vivo verteporfin treatment showed its ability to suppress hepatomegaly caused by YAP overexpression or activation of endogenous YAP. The anti-cancer effects of verteporfin were further documented in bladder cancer [137], pancreatic cancer [138,139], ovarian cancer [140], breast cancer [141] and melanoma [142]. The prospective anti-cancer use of verteporfin is also supported by its ability to synergically enhance the efficacy of other drugs. Verteporfin can reverse paclitaxel resistance in colorectal cancer and TNBC in a YAP-dependent manner [143,144,145]. In HER2-positive breast cancer, verteporfin diminishes lapatinib resistance caused by extracellular matrix rigidity [74]. Correspondingly, verteporfin in combination with a platinum compound (cisplatin or carboplatin) or paclitaxel show synergistical cytotoxicity in ovarian cancer cells [146]. Verteporfin also induces significant death of gemcitabine-nonresponsive pancreatic cancer cells by elevating the BAX/Bcl-xL ratio toward a pro-apoptotic balance [147]. Verteporfin exerts its cytotoxic function not only through inhibiting the oncogenic activity of YAP, but also triggering tumor-specific lysosomal membrane permeabilization (LMP), which provokes intracellular catabolic dysregulation and proteotoxicity [148]. Verteporfin-induced LMP potentiates the anti-cancer effect of sorafenib (a first-line systemic treatment in HCC) and overcomes its acquired resistance in HCC [148]. 

### 4.2. Dobutamine

Nucleocytoplasmic distribution of YAP/TAZ regulated by the Hippo pathway marks the essential molecular event of this evolutionarily conserved signaling process. Through monitoring the subcellular localization of YAP in cells treated with various chemical compounds, Bao and colleagues identified dobutamine, an agonist of β-adrenergic receptor, as an effective agent for inducing cytoplasmic accumulation of YAP [172]. Dobutamine induces phosphorylation of YAP-Ser127 and subsequent suppression of YAP-dependent gene transcription [172,173] (Figure 2 and Table 2). Dobutamine is guideline-recommended as one of the first-line medications for the treatment of acute heart failure [174,175], and is also used in cancer patients complicated with congestive heart failure [176] and cardiogenic shock [177]. Recently, its potential anti-cancer effect was examined in several cancer types. Human osteosarcoma cells following dobutamine treatment showed attenuated cell growth, invasiveness and migration, as well as augmented apoptosis [149]. Moreover, dobutamine significantly enhances apoptosis of gastric adenocarcinoma cells by sequestering phosphorylated YAP in the cytosol [150]. Nevertheless, in order to complement this research and add greater clinical relevance to the anti-cancer use of dobutamine, more studies are necessary to characterize its efficacy and potency.

### 4.3. Forskolin and Phosphodiesterase Inhibitors

Among the upstream regulators of the Hippo pathway, GPCR signaling acts in a particularly complex way in which it can either activate or inhibit the Hippo pathway dependent on the coupled G protein [44]. Stimulation of Gs-coupled receptors and subsequent production of the downstream cyclic adenosine monophosphate (cAMP) are able to activate LATS1/2 kinase activity and ultimately inhibit YAP function through protein kinase A (PKA) and Rho GTPases [153].Accordingly, forskolin, an adenylyl cyclase activator which leads to the production of cAMP, can significantly increase YAP phosphorylation and cytoplasmic accumulation [44] (Figure 2 and Table 2). Despite the fact that forskolin has been used in traditional medicine for centuries, this plant-produced labdane diterpene is basically utilized as a biological tool for cAMP elevation, other than a common therapeutic agent [151]. However, numerous studies have provided conceivable reasons for the clinical benefit that patients with various disorders, including cancer, might obtain from therapeutics containing forskolin. For example, forskolin in combination with rolipram (a selective phosphodiesterase 4 (PDE4) inhibitor) causes significant growth arrest and cell death in chemoresistant colon cancer cells [152]. Forskolin also suppresses rhabdomyosarcoma growth in mouse xenograft models [178]. However, whether the mechanisms of LATS1/2 activation and YAP inhibition caused by forskolin can explain its potential anti-cancer effect remains to be examined.

Opposite to cAMP production, cAMP can be degraded by phosphodiesterases (PDEs), which play a critical role in controlling the total amount of cAMP (as well as other cyclic nucleotides, for example, cyclic guanosine monophosphate (cGMP)) and creating the subcellular compartmentalization of cyclic nucleotide signaling [179]. Emerging evidence supports that PDE inhibitors are attractive therapeutic strategies for the treatment of Alzheimer’s disease, respiratory diseases, cancers, and many other illnesses. In line with the effect of forskolin on cAMP increase, PDE Inhibitors, which include theophylline, IBMX (both are nonselective PDE inhibitors), ibudilast (a PDE4 selective inhibitor), and rolipram, can induce YAP phosphorylation by restricting cAMP breakdown [153], suggesting the PDE inhibitors may be useful in the treatment of cancer types driven by YAP oncogenic activity (Figure 2 and Table 2).

Interestingly, another study further supports the clinical application prospects of PDE inhibitors in cancer treatment [154]. This study shows that PDE5/cGMP/protein kinase G (PKG) signaling maintains stemness of prostate cancer stem cells (PCSCs) through the hippo/TAZ pathway. Both PDE5 and TAZ are highly expressed in PCSCs and TAZ knockdown or PDE5 inhibition by its specific inhibitors (vardenafil or endogenous nitrogen monoxide (NO)) enables the attenuation of stemness in PCSCs, such as elevated cisplatin cytotoxicity [154].Inhibition of PDE5 activates PKG and subsequently MST/LATS kinase activities, which induce TAZ phosphorylation and cytosolic degradation [154]. Taken together, given the effectiveness of PDE inhibitors in restricting YAP/TAZ oncogenic activities, PDE inhibitors in combination with other medicines could potentially offer advanced anti-cancer strategies. 

### 4.4. Mevalonate Pathway Inhibitors

The interplay between the mevalonate pathway and hippo pathway was discovered through fluorescence-microscopy-based high-throughput screening of an FDA-approved drug library [155]. This research found that five statins, including cerivastatin and simvastatin, had the strongest YAP/TAZ inhibitory effect among the screened compounds (Figure 2 and Table 2). These observations are in agreement with a recent study in which a similar high-throughput small-molecule screening for YAP inhibitors was performed on primary lung fibroblasts [180]. Statins are a class of compounds commonly used to decrease serum cholesterol levels in patients with hypercholesterolemia. They function through suppressing hydroxymethylglutaryl-coenzyme A (HMG-CoA) reductases, the rate-limiting enzymes in the synthesis of a fatty acid intermediate named mevalonate [181]. The inhibition of mevalonate production by statins attenuates YAP/TAZ activities, which require mevalonate and its downstream geranylgeranyl pyrophosphate (GGPP) and Rho GTPases in a LATS1/2-independent manner [155]. Apart from the above mechanism, simvastatin promotes YAP phosphorylation at Ser127 in breast cancer cells, eventually leading to the cytoplasmic retention and degradation of YAP [156]. In HCC cells, simvastatin and fluvastatin were found to regulate the expression and phosphorylation of TAZ [157]. Strikingly, these results support the consideration of using statins as a new approach in cancer prevention and treatment. Indeed, a range of retrospective studies have demonstrated that statin use in cancer patients is significantly associated with lower cancer-related mortality [158,159,160]. These analyses inspire plans for clinical trials to further comprehensively evaluate the anti-cancer efficacy of statins. Indeed, several clinical trials (ClinicalTrials.gov: NCT03872388; NCT0345529) are ongoing to test how well statins function in cancer treatment.

### 4.5. Peptide Mimicking VGLL4 

Vestigial-like protein VGLL4 is a transcriptional repressor that directly competes with YAP for TEADs binding through its Tondu (TDU) domains [162,163]. It is downregulated and acts as a tumor suppressor in various cancer types [161,162,163]. The TDU domains of VGLL4 alone are sufficient for inhibiting YAP activity and, based on this property, a peptide designated “Super-TDU” was rationally invented to target YAP-TEADs interaction [162] (Figure 2 and Table 2). Through functionally mimicking VGLL4 and subsequently blocking YAP activity, this peptide potently suppresses gastric cancer growth in vitro and in mouse models, including nude mice bearing gastric cancer cells and *H. pylori*-infected mice [162]. Super-TDU treatment of adenomatous polyposis coli (*APC*) mutant mice (*APCmin/+*) (a mouse model typically used for colorectal and intestinal cancer) also significantly reduces the number and size of spontaneous adenomas without exhibiting detectable toxicity [161]. In pancreatic cancer, the disruption of YAP transcriptional activity by Super-TDU suppresses secreted YAP/TEAD target genes implicated in extracellular matrix remodeling, such as *AREG*, *CTGF*, *CYR61*, *fibroblast growth factor 1 (FGF1)* and *mesothelin (MSLN)* [164]. Given the fact that extracellular matrix remodeling and stiffening serve as critical factors of cancer aggressiveness [165,166], underlying the exact effect of Super-TDU on the tumor microenvironment could enhance its clinical application possibility. Taken together, these findings indicate this VGLL4-mimicking peptide which blocks YAP-TEAD interaction might be an effective tool in treating YAP-driven cancers.

### 4.6. Tyrosine Kinase Inhibitors

Tyrosine kinase inhibitors represent a tremendously important group of anti-cancer agents. Dasatinib is a dual BCR/ABL and Src family tyrosine kinase inhibitor approved for chronic myelogenous leukemia (CML) and acute lymphoblastic leukemia (ALL) treatment. Dasatinib inhibits the nuclear accumulation of YAP and impedes the formation of β-catenin-YAP-T-Box transcription factor 5 (TBX5) complex via inhibiting the phosphorylation of YAP-Tyr357 by tyrosine kinase YES1 (Figure 2 and Table 2) [167]. Diverse studies have proved the potent anti-cancer effect of dasatinib through inhibiting YAP activity in colon cancer, renal cell carcinoma, pancreatic cancer and other cancer types [167,168,169].

### 4.7. CDK9 Inhibitor and BET Inhibitor

Cyclin-dependent kinase 9 (CDK9) is the catalytic subunit of the positive transcription elongation factor b (P-TEFb) and phosphorylates the carboxyl-terminal domain (CTD) of RNA polymerase II (RNA-Pol II) [182]. Cancer cells evolve an intensive dependency on dysregulated transcriptional machinery, driven by their uncontrolled proliferation or other malignant behaviors, which is recently defined as transcriptional addiction [171]. Therefore, the hypothesis that CDK9 could be an anti-cancer therapeutic target has been proposed and are under investigation, based on its pivotal role in transcription elongation. The mechanistic studies on the role of CDK9 in YAP-driven tumorigenesis gradually unveiled the mystery of how YAP/TAZ-TEAD regulates gene transcription. In cancer cells, YAP/TAZ tends to predominantly promote the transcription of their target genes by recruiting Mediator complex to the enhancers, and this further recruits CDK9, thus activating transcription elongation of YAP/TAZ target genes implicated in tumorigenesis (Figure 2 and Table 2) [170]. CDK9 blockade by using the inhibitors (Flavopiridol and NVP-2) is able to suppress the target genes expression and YAP-driven liver overgrowth in mice [170]. These discoveries imply that pre-clinical and clinical studies might consider the suppression of YAP/TAZ-driven transcription via CDK9 inhibitors. 

In addition, another study points out Bromodomain-containing protein 4 (BRD4), a general regulator of RNA-Pol II-dependent transcription, is recruited to the promoter of YAP/TAZ target genes by YAP/TAZ-bound enhancers, thus boosting the transcription of genes involved in tumorigenesis [171]. Hence, this transcriptional addiction mediated by YAP/TAZ can be blocked by Bromodomain and Extra-Terminal motif (BET) protein inhibitors (such as JQ1), even when YAP is overexpressed (Figure 2 and Table 2) [171]. JQ1 not only potently impairs tumor cell proliferation, cell transformation and in vivo tumor growth in YAP/TAZ-addicted breast, liver and pancreatic cancer models; but also sensitizes BRAF-mutant melanoma cells to vemurafenib treatment [171]. These data offer the potential use of BET inhibitors in targeting YAP/TAZ to revert drug resistance. 

## 5. Conclusions

Hyperactivation of YAP/TAZ, or loss-of-function of tumor suppressors in the Hippo pathway is able to mediate tumor cell resistance against anti-cancer therapeutic drugs. Depletion or inhibition of YAP/TAZ through pharmacological agents, or re-expression of tumor suppressors in the Hippo pathway reverts the drug sensitivity, suggesting intervention of YAP/TAZ activity is a prospective strategy to overcome drug resistance in cancer cells. 

## 6. Future Perspectives

In addition to controlling cell growth and apoptosis, the Hippo pathway is also implicated in drug resistance, as documented in a variety of cancer types. Studies indicate significant superiority of pharmacologically targeting the Hippo pathway, particularly YAP/TAZ, over current agents to eliminate cancer cells. However, several critical questions need to be answered for successful future translation of these preclinical studies into powerful clinical strategies. Given that targeting the Hippo pathway shows attractive perspectives in anti-cancer treatment, well-designed clinical trials will be necessary for the successful practice of evidence-based health care. Indeed, FDA-approved non-anti-cancer drugs, such as verteporfin, simvastatin and ibudilast, are under active clinical trials to evaluate their effectiveness in cancer therapy. Considering the reasonable safety of these medicines that have been applied in clinic for many years, their paths to be incorporated into standard cancer treatment would presumably be much shorter than newly invented compounds. This scenario also applies to FDA-approved anti-cancer agents, for example, specific kinase inhibitors that suppress YAP/TAZ activities. Decipher of these mechanisms broaden the scope of the drug applications in cancer treatment, supplying more medications for the cancer types which are relevantly rare and have limited therapeutic options. However, it should be underlined that simply relying on existing drugs or chemicals is not sufficient to solve the drug resistance problems. Inventions of novel compounds with elevated anti-cancer efficiency, substrate specificity and attenuated adverse effects are in demand. For example, although BET inhibitors significantly enhance the vulnerability of melanoma cell under RAF inhibitor treatment, selective BET inhibitors, especially inhibitors selectively targeting BRD4, are scarcely available. In-depth illustration of the protein quaternary structure, protein-to-protein interactions and structure dynamics might advance the design of improved inhibitors.

In addition, clinical trials will provide feasible methods for oncologists to select the patients who are thought most likely to benefit from the treatment, which could be generally explained as the maximum of drug efficacy and the minimum of unfavorable clinical outcomes. Although YAP/TAZ are frequently activated in various cancers, the efficacy of YAP/TAZ-targeted treatment and the possible side effects may vary from patient to patient, which might require a personalized therapy for each individual. Specific biomarker tests (for the presence of proteins, gene expression, specific cells etc.) appear to represent the most common method to predict drug response, which also need to be investigated to anticipate the outcomes of YAP/TAZ-targeted therapy. And cohort studies are also valuable strategies to stratify patients depending on their shared characteristics. 

It should be pointed out, in contradiction to studies defining YAP as an oncogene, some studies also showed a tumor suppressive function for YAP. In breast cancer, loss of YAP protects cancer cells from anoikis, and YAP silencing promotes cancer cell migration and invasion [183]. YAP knockdown enhances cell proliferation, migration and protection from cisplatin-induce cell death in head and neck cancer cells [55]. YAP activation was found to attenuate gemcitabine resistance in pancreatic cancer cells [119]. Moreover, overexpression of YAP pronouncedly increases sensitivity of HCC cells to cisplatin and doxorubicin [59]. Importantly, the tumor suppressive roles of YAP tend to be regulated by p53 family members, including p53 and p73, and a hypothesis that p53 status serves as the molecular switch to determine the function of YAP (either as an oncogene or a tumor suppressor) has been proposed [184]. Increasing attention must be paid to delineate the molecular architecture that dictate YAP/TAZ functions, either as an oncogene or a tumor suppressor. This clarification is pivotal especially when clinicians select the optimal anti-cancer treatments for patients and YAP/TAZ-targeted therapies are considered. Moreover, such an advance will help profile signaling pathways implicated in drug resistance and accelerate rational development of anti-cancer treatments harboring better response coupled with lower side effects. 

Over the past years, although enormous efforts have been made to identify the complicated functions, regulations, and networks of the Hippo pathway, the underlying mechanisms how this signaling drives drug resistance remain largely obscure. YAP/TAZ hyperactivity or overexpression is apparently not the ultimate answer to this question. What are the downstream genes induced by YAP/TAZ and then more directly implicated in drug resistance? How does the Hippo pathway collaborate with other signaling to regulate YAP/TAZ activation in driving drug resistance? These mechanisms may clue more powerful multi- drug combinations in terms of further beneficial therapeutics for cancer patients. Moreover, stemness and microenvironment-directed drug resistance are two critical features of malignant cells, but relatively less investigated. What is the influence of YAP/TAZ-targeted treatment in cancer stem cell biology and tumor microenvironment? Deciphering these genetic, molecular, and cellular players related to YAP/TAZ biology will elicit more innovations in anti-cancer treatment and define more potentials of YAP/TAZ-target therapy.

## Figures and Tables

**Table 1:** Summary of the Hippo components implicated in resistance to anti-cancer drug.

Hippo Components	Dysregulation	Anti-Cancer Drug	Cancer Type	Reference
YAP	Hyperactivation or overexpression	Paclitaxel	Ovarian cancer	[53]
		Cisplatin	Ovarian cancer and HNSCC	[54,55]
		Doxorubicin	HCC	[56,57,58,59]
		5-Fluorouracil	Colorectal cancer	[60,61,62]
		Gemcitabine	Pancreatic cancer, TNBC and gallbladder cancer	[63,64,65,66,67]
		EGFR-TKIs	Lung cancer, ovarian cancer and colorectal cancer	[68,69,70,71,72,73]
		Lapatinib	Breast cancer	[74]
		CDK4/6 inhibitors	Breast cancer	[75]
		RAF inhibitors	NSCLC and melanoma	[76,77]
		MEK inhibitors	NSCLC and neuroblastoma	[76,78,79]
TAZ	Hyperactivation or overexpression	Paclitaxel	Breast cancer	[80,81,82,83]
		Cisplatin	Nasopharyngeal carcinoma and prostate cancer	[84,85]
		Doxorubicin	Breast cancer and HCC	[56,57,58,82]
		Lapatinib	Breast cancer	[74]
		CDK4/6 inhibitors	Breast cancer	[75]
MST1	Downregulation	Cisplatin	Prostate cancer	[85]
LATS1/2	Downregulation	Paclitaxel	Cervical cancer, ovarian cancer and NSCLC	[86,87,88]
		5-Fluorouracil	Colorectal cancer	[89]
RASSF1A	Epigenetically silencing	Paclitaxel	Ovarian and breast cancer	[54,90,91]
hEx	Downregulation	Paclitaxel	Breast cancer	[92]
MYPT1	Downregulation	Cisplatin	Ovarian cancer	[93]
Ajuba	Overexpression	Cisplatin	Cervical cancer	[94]
RASSF6	Downregulation	Doxorubicin	Bladder cancer	[95]
Merlin	Downregulation	5-Fluorouracil	Glioma	[96]

**Table 2:** Summary of chemicals targeting the Hippo signaling pathway.

Agent	Mechanism	Cancer Type	Treatment Outcome	Reference
Verteporfin	Inhibit YAP-TEADs interaction	Bladder cancer, pancreatic cancer, ovarian cancer, breast cancer, melanoma and HCC	Increase cytotoxicity of other anti-cancer drugs	[75,138,139,140,141,142,143,144,145,146,148,149,150]
Dobutamine	Induce YAP cytoplasmic retention	Osteosarcoma and gastric adenocarcinoma	Enhance cell apoptosis	[149,150]
Forskolin	Increase YAP phosphorylation and cytoplasmic accumulation	Colon cancer and rhabdomyosarcoma	Attenuate tumor growth and promote cell death	[44,151,152]
PDEs	Increase YAP phosphorylation and cytoplasmic accumulation	Prostate cancer	Elevated cisplatin cytotoxicity	[153,154]
Mevalonate pathway inhibitors	Promote YAP/TAZ phosphorylation, cytoplasmic retention and degradation	HCC, breast cancer and ovarian cancer	Associated with lower cancer-related mortality	[155,156,157,158,159,160]
Super-TDU	Compete with YAP for TEADs binding	Gastric cancer and pancreatic cancer	Suppress tumor growth	[161,162,163,164,165,166]
Tyrosine kinase inhibitors	Inhibit YAP nuclear accumulation	Colon cancer, renal cell carcinoma, pancreatic cancer	Suppress tumor growth	[167,168,169]
CDK9 inhibitor	Block YAP/TAZ-mediated transcription	Liver cancer	Suppress tumor growth	[170]
BET inhibitor	Block YAP/TAZ-mediated transcription	Breast cancer, liver cancer, pancreatic cancer and melanoma	Suppress tumor growth and enhance the cytotoxicity of RAF inhibitor	[171]
